# Supplementary material for: Serological testing of blood donors to characterise the impact of COVID-19 in Melbourne, Australia, 2020
Source: PLoS One. 2022 Jul 6;17(7):e0265858. doi: 10.1371/journal.pone.0265858 (PMC9258843; doi:10.1371/journal.pone.0265858)
Supplement: S1 Table — (DOCX) [file pone.0265858.s001.docx]

**S1 Table. List of postcodes, by sampling group**

| **Low incidence**  **<3 cases/1,000 population** | **Medium incidence**  **3-7 cases/1,000 population** | **High incidence**  **>7 cases/1,000 population** |
| --- | --- | --- |
| 3071 | 3006 | 3003 |
| 3088 | 3012 | 3004 |
| 3103 | 3013 | 3011 |
| 3105 | 3028 | 3019 |
| 3106 | 3036 | 3020 |
| 3115 | 3039 | 3021 |
| 3116 | 3042 | 3022 |
| 3125 | 3048 | 3023 |
| 3126 | 3054 | 3024 |
| 3127 | 3055 | 3025 |
| 3129 | 3056 | 3026 |
| 3131 | 3057 | 3027 |
| 3133 | 3059 | 3029 |
| 3135 | 3068 | 3031 |
| 3142 | 3072 | 3034 |
| 3146 | 3093 | 3037 |
| 3153 | 3143 | 3046 |
| 3154 | 3175 | 3047 |
| 3161 | 3177 | 3051 |
| 3162 | 3181 | 3052 |
| 3167 | 3186 | 3060 |
| 3173 | 3335 | 3061 |
| 3178 | 3805 | 3064 |
| 3183 | 3809 | 3065 |
| 3184 | 3975 | 3066 |
| 3193 | 3976 | 3074 |
| 3197 | 3977 | 3076 |
| 3198 |  | 3081 |
| 3201 |  | 3090 |
| 3204 |  | 3336 |
| 3205 |  | 3337 |
| 3428 |  | 3338 |
| 3800 |  | 3427 |
| 3807 |  | 3750 |
| 3911 |  | 3753 |
|  |  | 3803 |
|  |  | 3978 |
