# Supplementary material for: Serological testing of blood donors to characterise the impact of COVID-19 in Melbourne, Australia, 2020
Source: PLoS One. 2022 Jul 6;17(7):e0265858. doi: 10.1371/journal.pone.0265858 (PMC9258843; doi:10.1371/journal.pone.0265858)
Supplement: S1 File — (DOCX) [file pone.0265858.s002.docx]

**S1 File: Multilevel regression and poststratification modelling**

The following multilevel model, including main effects for postcode, sampling stratum, sex, age and socioeconomic quintile, was specified for $\pi_{i}$, the true probability of SARS-CoV-2 total antibody present for specimen *i*:

$\pi_{i}=\mathrm{logit}^{-1}\left( \beta_{1}+\beta_{2}\mathrm{male}_{i}+\beta_{3}\mathrm{SES}_{i}+\alpha_{age[i]}^{\mathrm{age}}+ \alpha_{SES[i]}^{\mathrm{SES}}+\alpha_{pcode[i]}^{\mathrm{pcode}}+\alpha_{strata[i]}^{\mathrm{strata}} \right)$,

where, male is a variable that takes on the value of 0 for women and 1 for men, SES represents socioeconomic quintiles measured at the postcode-level, age[i], SES[i], sex[i], pcode[i] and strata[i] are index variables for specimen i, $\beta_{1}$, $\beta_{2}$ and $\beta_{3}$ are logistic regression coefficients and the α parameters are vectors of varying coefficients (random effects). These coefficients have hierarchical priors as follows:

$$\alpha^{\mathrm{age}}\sim normal\left( 0, \sigma^{\mathrm{age}} \right)$$

$$\alpha^{\mathrm{SES}}\sim normal\left( 0, \sigma^{\mathrm{SES}} \right)$$

$$\alpha^{\mathrm{pcode}}\sim normal\left( 0, \sigma^{\mathrm{pcode}} \right)$$

$$\alpha^{\mathrm{strata}}\sim normal\left( 0, \sigma^{\mathrm{strata}} \right)$$

Prior distributions for model parameters were specified as per Gelman & Carpenter (2020). That is, $\sigma^{\mathrm{age}}$, $\sigma^{\mathrm{SES}}$,$\sigma^{\mathrm{pcode}}$,$\sigma^{\mathrm{strata}}$ were assigned normal^+^(0,0.5) priors, which allows the prevalence to vary moderately by these covariates. In the primary analysis, a unit logistic prior was defined for the centred intercept $\beta_{1}+\beta_{2}\bar{\mathrm{male}}+\beta_{3}\bar{\mathrm{SES}}$ (where $\bar{\mathrm{male}}$ and $\bar{\mathrm{SES}}$ represent the proportion male and the mean SES in the sample, respectively). This corresponds to a flat uniform (0, 1) prior distribution for the probability that an average person in the sample has the antibody. In sensitivity analysis, an alternative logistic (–3.5,1) prior distribution was specified, which focuses on values for seroprevalence below 5% (prior probability prevalence less than 2% and less than 5% were 0.40 and 0.64 respectively).

The following expression defines the observed prevalence $p$, given the true prevalence $\left( \pi\right)$ and test sensitivity $\left( \delta\right)$ and specificity $\left( \gamma\right)$:

$p=\pi\delta+\left( 1-\pi\right)\left( 1-\gamma\right)$.

Poststratification adjustment was used to produce an estimate of prevalence for the Melbourne metropolitan blood donor population from 2019 (*N* = 29,731; see Table 1 for summary of basic demographics) as well as the Melbourne metropolitan resident population aged 20–69 years (*N* = 2,678,532; Table 1)
