# Supplementary material for: Serological testing of blood donors to characterise the impact of COVID-19 in Melbourne, Australia, 2020
Source: PLoS One. 2022 Jul 6;17(7):e0265858. doi: 10.1371/journal.pone.0265858 (PMC9258843; doi:10.1371/journal.pone.0265858)
Supplement: Attachment — Submitted filename: PONE-D-22-06787 Responses.docx [file pone.0265858.s004.docx]

**PONE-D-22-06787: Serological testing of blood donors to characterise the impact of COVID-19 in Melbourne, Australia, 2020**

Journal Requirements:

When submitting your revision, we need you to address these additional requirements.

1. Please ensure that your manuscript meets PLOS ONE's style requirements, including those for file naming. The PLOS ONE style templates can be found at

<https://journals.plos.org/plosone/s/file?id=wjVg/PLOSOne_formatting_sample_main_body.pdf> and

<https://journals.plos.org/plosone/s/file?id=ba62/PLOSOne_formatting_sample_title_authors_affiliations.pdf>

**Response:** The manuscript has been formatted to the journal style requirements.

2. Please provide additional details regarding participant consent. In the ethics statement in the Methods and online submission information, please ensure that you have specified (1) whether consent was informed and (2) what type you obtained (for instance, written or verbal, and if verbal, how it was documented and witnessed). If your study included minors, state whether you obtained consent from parents or guardians. If the need for consent was waived by the ethics committee, please include this information.

If you are reporting a retrospective study of medical records or archived samples, please ensure that you have discussed whether all data were fully anonymized before you accessed them and/or whether the IRB or ethics committee waived the requirement for informed consent. If patients provided informed written consent to have data from their medical records used in research, please include this information.

**Response:** The ethics statement containing all relevant information has been included.

3. We note that the grant information you provided in the ‘Funding Information’ and ‘Financial Disclosure’ sections do not match.

When you resubmit, please ensure that you provide the correct grant numbers for the awards you received for your study in the ‘Funding Information’ section.

**Response:** The information has now been updated. The study was funded by two organisations: The Victorian Government Department of Health (no grant number) and Snow Medical Foundation (CT28701/G207593).

4. Thank you for stating the following in the Acknowledgments Section of your manuscript:

“The study was supported by the Victorian Government Department of Health and Snow Medical Foundation (CT28701/G207593). The Australian government funds Australian Red Cross Lifeblood to provide blood, blood products and services to the Australian community.”

We note that you have provided additional information within the Acknowledgements Section that is not currently declared in your Funding Statement. Please note that funding information should not appear in the Acknowledgments section or other areas of your manuscript. We will only publish funding information present in the Funding Statement section of the online submission form.

Please remove any funding-related text from the manuscript and let us know how you would like to update your Funding Statement. Currently, your Funding Statement reads as follows:

“The funders have no role in study design, data collection and analysis, decision to publish, or preparation of the manuscript.”

Please include your amended statements within your cover letter; we will change the online submission form on your behalf.

**Response:** Many thanks, the information has been provided in the cover letter.

5. In your Data Availability statement, you have not specified where the minimal data set underlying the results described in your manuscript can be found. PLOS defines a study's minimal data set as the underlying data used to reach the conclusions drawn in the manuscript and any additional data required to replicate the reported study findings in their entirety. All PLOS journals require that the minimal data set be made fully available. For more information about our data policy, please see <http://journals.plos.org/plosone/s/data-availability>.

Upon re-submitting your revised manuscript, please upload your study’s minimal underlying data set as either Supporting Information files or to a stable, public repository and include the relevant URLs, DOIs, or accession numbers within your revised cover letter. For a list of acceptable repositories, please see <http://journals.plos.org/plosone/s/data-availability#loc-recommended-repositories>. Any potentially identifying patient information must be fully anonymized.

Important: If there are ethical or legal restrictions to sharing your data publicly, please explain these restrictions in detail. Please see our guidelines for more information on what we consider unacceptable restrictions to publicly sharing data: <http://journals.plos.org/plosone/s/data-availability#loc-unacceptable-data-access-restrictions>. Note that it is not acceptable for the authors to be the sole named individuals responsible for ensuring data access.

We will update your Data Availability statement to reflect the information you provide in your cover letter.

**Response:** A minimum underlying data set has been uploaded with the re-submission.

6. Please review your reference list to ensure that it is complete and correct. If you have cited papers that have been retracted, please include the rationale for doing so in the manuscript text, or remove these references and replace them with relevant current references. Any changes to the reference list should be mentioned in the rebuttal letter that accompanies your revised manuscript. If you need to cite a retracted article, indicate the article’s retracted status in the References list and also include a citation and full reference for the retraction notice.

**Response:** The references list has been checked and updated.

**Review Comments to the Author**

Reviewer #1: The manuscript SEROLOGICAL TESTING OF BLOOD DONORS TO CHARACTERISE THE IMPACT OF COVID-19 IN MELBOURNE, AUSTRALIA, 2020, with good sampling strategy, estimated SARS-Cov-2 specific antibody prevalence among blood donors in metropolitan Melbourne following a COVID-19 outbreak in the city during summer of 2020. This study provides a surveillance measurement to monitor seroprevalence and infection rates , track outbreaks within communities. As mentioned in the DIDCUSSION, This study has some limitation:
1. Blood donors represents a population less likely to be infected by SARS-CoV-2 than general population;
2. They are normally in better health status to pass the recruitment criteria;
Therefore the test results mostly represent asymptomatic cases. and
3. Antibodies from asymptomatic individuals are usually weaker., which requires higher sensitivity of the assay.
4. Blood donor population doesn't represent people under 16 years, and more and more cases are reported from this age group generally.
Overall, This is a good research study, which reflects the pattern and trends of SARS-Cov-2 infection in metropolitan Melbourne during the outbreak in summer of 2020. Due to the limitation of the selected population, the extent of infection spread and proportion to reported cases of infection could be underestimated. Hope authors can revise their study aim statements.

**Response:** We have made small changes to clarify that the aim of the study was to measure seroprevalence among blood donors to compare with notifications in Melbourne.

Reviewer #2: This is an interesting paper looking at serological testing of blood donors to characterize the prevalence of covid-19 from another perspective. Prevalence of infection is related to different status and demographic groups. However, a few remarks:

- In Fig 1: before starting vaccination program

**Response:** Paragraph 1 of the materials and methods (last sentence), has been updated with the above detail.

- Results: (Paragraph 1) What is the p value?

**Response:** The aim of the study was to explore the association between seroprevalence and case notifications and not to formally compare seroprevalence between the three sampling groups which were utilised to inform the sampling method. As such, paragraph 1 which describes the cohort characteristics presented in Table 1, should be interpreted from this perspective.

- Results: (Paragraph 2) Please show the results in table.

**Response:** The results described in paragraph 2 were presented in Supplementary Table 2. This table has been moved to the main body of the manuscript.

- Figure 2 is not clear.

**Response:** New versions of the figures have been uploaded with the submission.

- Please consider the conclusion of study.

**Response:** We agree with the reviewer that the conclusion was somewhat disjointed. We have made some changes in the final discussion paragraphs to better integrate the concluding statements with the study findings.
